# The association between crop diversity and children’s dietary diversity: multi-scalar and cross-national comparisons

**DOI:** 10.1007/s12571-024-01458-9

**Published:** 2024-07-03

**Authors:** Carina Isbell, Daniel Tobin, Brian C. Thiede, Kristal Jones, Travis Reynolds

**Affiliations:** 1 The University of Vermont, Burlington, VT, USA; 2 The Pennsylvania State University, University Park, PA, USA; 3 JG Research and Evaluation, Bozeman, MT, USA

**Keywords:** Crop diversity, Dietary diversity, Nutrition, Spatial scale

## Abstract

Food insecurity is rising across sub-Saharan Africa (SSA), where undernourishment continues to affect a large portion of the population, particularly young children. Studies examining the associations between crop diversity and childhood nutrition have recently proliferated but are characterized by inconsistent results and two key limitations. First, many studies focus only on the household level, overlooking the prospect that more diverse crops at village and regional levels may contribute to household food security. Second, many studies pool data from multiple countries, which may obscure important context-specific aspects of nutrition outcomes. Drawing on Demographic and Health Survey (DHS) data from 10 SSA countries, in combination with agricultural production estimates for 112 crop species, this study explores the associations between crop diversity at multiple scales (10-, 25-, and 50-kilometer radii) and children’s dietary diversity (HDDS). In addition to producing overall estimates across our sample, we measure country-specific associations to account for spatial heterogeneity. Results of the overall model show a negative association between crop diversity and dietary diversity. However, the country-specific analyses uncover extensive variability in these associations: in some cases, diversity is highly positively correlated with HDDS, while in others the estimated effect is negative or nonexistent. Our findings suggest that country-level analyses provide important nuance that may be masked in pooled analyses. Moreover, these findings foreground the importance of looking beyond household-level analyses to understand the dynamic role that local crop diversity, and its exchange across space, can play in supporting children’s dietary diversity.

## 1 Introduction

Current threats to global food security are extensive and continue to push international development targets, such as the United Nations Sustainable Development Goals (SDGs) of “Zero Hunger” and “Good Health and Wellbeing” by 2030, off-track. After half a decade of relative consistency, the prevalence of undernourished people worldwide rose to 9.9% in 2020 – an increase of 1.5 percentage points from the prior year (FAO et al., 2021). Although by 2022 those numbers had improved, there were an estimated 122 million more people experiencing hunger as compared to 2019 (FAO et al., 2023). Within the last few years, the COVID-19 pandemic has exacerbated food insecurity and specifically malnutrition, especially among children in low- and middle-income countries, where undernutrition continues to be a serious problem despite some progress within the last decade. Childhood undernutrition is particularly acute in subSaharan Africa (SSA) where between 30–35% of children are affected by stunting (Fanzo et al., 2017; Takele et al., 2022), and where conflict and environmental instability have converged to produce persistent food and nutritional crises throughout many countries (Anderson et al., 2021). Rural areas often face the brunt of food insecurity, with rural children more likely to experience undernutrition (e.g., stunting and micronutrient deficiencies) than their urban counterparts (FAO et al., 2022). Due to geographic isolation and disproportionately high rates of poverty (OECD/FAO, 2016)^1^, as well as other important contextual factors such as low production diversity and limited access to markets (Fraval et al., 2019), children living in rural households comprise a population particularly vulnerable to undernutrition, and continue to need evidence-based interventions to help improve their food and nutrition security.

Rural households in SSA overlap significantly with small-scale farm households. According to a recent World Bank study of nine SSA countries, 98% of households in rural areas are involved in agriculture at least to some degree (Christiaensen & Demery, 2018). Yet despite small-scale farm households producing approximately one-third of the world’s food (Lowder et al., 2021), they are also among the most food insecure globally (FAO et al., 2022). For this reason, emphasis has been placed on improving food and nutrition security among these populations, and particularly for children under the age of five years old. Improving the food security of young children entails enhancing both the caloric adequacy and macro- and micro-nutrient content of the foods they consume, which is particularly important for reducing the prevalence of stunting and wasting (Aboagye et al., 2021). Both outcomes are prevalent throughout SSA and are common manifestations of undernutrition. Undernutrition, which relates both to not having enough food *and* the insufficient availability of nutrients, during this vital stage of development puts children at high risk for diet-related noncommunicable diseases, mortality, and lifelong health challenges (Hoddinott et al., 2013; Akombi et al., 2017; Willet et al., 2019; Clark et al., 2020). Increasing the variety of foods consumed (i.e., dietary diversity) is one method that can help address undernutrition among children (Zeinalabedini et al., 2023), and concurrently enhance micronutrient adequacy (Leroy et al., 2015; Molani-Gol et al., 2023). Crop diversification is a commonly pursued strategy that can increase dietary diversity among rural children. Crop diversification can improve the health and well-being of small-scale farm households through several key pathways and has the potential to contribute to important environmental and climate resilience goals. Moreover, crop diversification can directly increase the food (and nutritional) diversity available to households (Jones, 2017a) while also creating new sources of income generated by entering new markets and helping small-scale farmers buffer against market and weather-related risks (Bellon et al., 2016).

However, despite the potential for crop diversification to improve food and nutrition security for small-scale farmers and their families, evidence has been mixed as to the practical impact crop diversification can have at the household level (Jones, 2017a; Sibhatu & Qaim, 2018). These results have led to disagreements about the usefulness of promoting crop diversification over alternative methods, such as specialization (i.e., focusing on a smaller set of market-oriented crops that can be integrated into regional and global value chains and generate income used to purchase food). That said, these disagreements concentrate on the efficacy of increasing *household* crop diversity to support *household* food and nutrition security – intentionally or unintentionally maintaining the assumption that food production and consumption principally operate as a closed loop within individual households of small-scale farmers. Despite the importance of food obtained outside the household (e.g., through local or regional markets and non-market exchanges) in supporting food security and dietary diversity (Fraval et al., 2019), there remains a surprisingly limited body of empirical evidence on the impacts of crop diversification beyond the household level (Remans, 2015).

In response to this lack of evidence, there has been renewed emphasis on the potential for crop diversification at village, regional, and national scales to influence nutritional and food security outcomes (Jones, 2017a; Mariani et al., 2021; Nicholson et al., 2021; Remans et al., 2015; Renard & Tilman, 2019; Tobin et al., 2019). However, it remains unclear as to which scale of crop diversification might be the most appropriate, or how the importance of scale may vary by country or geographic context (Khonje et al., 2022). Building upon Tobin et al.’s (2019) multi-country analysis of village-level crop diversity and children’s nutritional outcomes, we examine the nutrition consumption impacts of crop diversification at multiple scales across 10 SSA countries and produce both overall (i.e., pooled) and country-specific estimates. Critically, we extend Tobin et al.’s (2019) analysis by refactoring crop production data to multiple scales around clusters of households. These measures allow us to construct more precise and nuanced measures of local crop diversity that can help guide interventions seeking to understand if and at what scale crop diversification can improve the nutrition of children in small-scale farm households. In the following analysis, we combine data from the Demographic and Health Surveys Program (DHS) with high-resolution estimates of crop production to examine the association between crop diversity (Simpson’s Diversity Index (SDI)) at multiple scales (10-, 25-, and 50-km radii around household clusters) and dietary diversity among under-five children in SSA. Through this analysis, we provide insight into the impact of crop diversification on the nutrition of children within small-scale farm households in subSaharan Africa.

## 2 Background and conceptual framework

### 2.1 Crop diversification, dietary diversity, and nutrition

Crop diversity is believed to be an important component of nutritional security, especially among rural and resource-poor populations (Martin-Prével et al., 2015). Efforts to increase access to nutritious foods through crop diversification have generally shown positive results, whether through food grown for one’s own household (Kumar et al., 2018; Bellon, 2020) or by being able to access more nutritious food via local markets (Muthini et al., 2020). Relatedly, evidence suggests that crop diversity may be especially important for households that live in remote areas, where higher degrees of crop diversity has generally been associated with the most significant improvements in children’s nutrition outcomes (Ruel et al., 2018). Indeed, crop diversity may not only translate into improvements in dietary diversity, but it may also affect other nutritional and health outcomes (Bakhtsiyarava & Grace, 2021; Bellon et al., 2020; Bellows et al., 2020; Gitagia et al., 2019; Lachat et al., 2018; Tesfaye & Tirivayi, 2020; Whitney et al., 2018). For example, dietary diversity from growing more types of crops has been found to be significantly and positively associated with child growth indicators such as height-for-age and weight-forage (Arimond & Ruel, 2004; Jones, 2017a; Lovo & Veronesi, 2019), which are indicators of chronic undernutrition and short-term nutrition inadequacy, respectively. Importantly, low height-for-age and weight-for-age are correlated with increased risks of other childhood illnesses and mortality, as well as health and socioeconomic attainment over the course of a person’s life far beyond childhood (Black et al., 2013; Hoddinott et al., 2013; Victora et al., 2008). The implication is that dietary improvements due to crop diversification may have important and multi-dimensional impacts on children’s nutrition, health, and development.

Although the potential nutritional and health benefits of crop diversity have received considerable attention, there is notable variation in the association between household crop diversity and nutrition security across geographic and social contexts – and in some cases evidence of the nutritional benefits of crop diversification is marginal at best (Namulondo & Bashaasha, 2021). While crop diversity is often statistically associated with positive nutritional outcomes, the impacts are not always practically meaningful enough to affect health in a substantial way (Jones, 2017a; Sibhatu & Qaim, 2018). As Sibhatu and Qaim (2018) point out in their meta-analysis, the average marginal effect of crop diversification on dietary diversity is very small across the 45 papers they analyzed, such that increases in the number of crop species grown within some farms may have negligible changes in the number of food groups consumed. Moreover, increasing crop diversity does not always result in increasing the number or types of food within fields – for instance, especially within rural areas, households may have high overall crop diversity, but relatively low food crop diversity if they are also growing other crops they may need for medicinal, fiber, feed/fodder, or market purposes (Whitney et al., 2018). In addition, increased crop diversity might not enhance an income or livelihood pathway to improved dietary diversity if local markets are inadequate or lack demand for the diversified crop species, and/or if those local markets are not well-connected to into regional, national, or international markets (Jones, 2017a).

The associations between crop diversity and dietary diversity have also been found to vary significantly by country (Nandi et al., 2021), foregrounding how the effects of crop diversification on dietary intake may vary by context. For example, the success of interventions seeking to enhance dietary diversity through crop diversification may require concurrent efforts to enhance nutritional education, women’s empowerment, as well as the quality of water and sanitation infrastructure (Ruel et al., 2018). The variation in the impact of crop diversification is noteworthy: it suggests that not all households may benefit by increasing the number of species in their fields and gardens. However, considering that one household’s diversification can affect the consumption and nutrition patterns beyond its own fields (e.g., through sales, gifting, and other exchanges), this variation highlights the importance of looking beyond the household level to understand how crop diversity may benefit children across local to regional scales.

### 2.2 Crop diversity and nutrition beyond the household

Most existing research that has examined the intersections between agrobiodiversity and nutrition has focused on the household level (Fanzo, 2017). The possible effects of agrobiodiversity at scales beyond the household are understudied, despite theoretical and empirical bases for expecting local and regional patterns of crop production to affect individuals’ nutrition (Tobin et al., 2019). There are strong conceptual reasons to expect crop diversification at supra-household scales to positively affect nutritional outcomes. Perhaps most simply, few households rely entirely upon their own production. Even among a sample of rural subsistence-oriented farmers in East Africa studied by Remans et al. (2011), only about half of household food consumption was self-produced. This finding is consistent with other studies focused on low-income countries in SSA and globally (Jones, 2017a; Shikuku, 2019). Yet despite evidence that crop production beyond the household scale has the potential to impact household food and nutrition security among small-scale farm households, the impact of food sources outside of households is not often a considered in empirical studies (Fraval et al., 2019).

Social networks and informal exchanges of both seeds and food are particularly overlooked despite their ability to support the health and livelihoods of small-scale farm households (Jones, 2017b; Shikuku, 2019). As exemplified by Moges et al.’s (2022) study of rural communities in Ethiopia, a significant degree of the essential micronutrients (e.g., vitamin A, zinc) that children consume are contributed by local farming systems. This dynamic suggests that rural children rely on food obtained from outside the household but within their locality to fulfill essential micronutrient requirements. Analyses limited to the household level thus miss the importance of community context, which has been shown to be a strong predictor of both crop diversification and nutritional outcomes (Tesfaye & Tirivayi, 2020; Tobin et al., 2019).

In addition to the direct links between local crop diversity and food consumption, crop diversification may also improve food and nutritional security at wider scales than the household through economic mechanisms (Bellon et al., 2016; Sibhatu & Qaim, 2018). Previous research has highlighted how crop diversification may be most effective in enhancing smallholder nutrition outcomes when there are potential outlets for selling agricultural surplus and therefore diversifying sources of income (Rahman & Chima, 2016). Although the most profitable outlets are often considered to be those outside the areas in which crops are grown, crop diversification in areas where there previously was very little crop diversity can increase local demand as well – meaning that diversifying the fields of even a few households in some communities can generate local demand and thus consumption, especially in extremely rural areas (Jones, 2017a). Such changes in demand may allow for the possibility of markets to flourish in areas they may not have before because of low diversity or a high reliance on exporting goods outside of communities rather than keeping them within. Indeed, considering that the strength of association between crop diversity and nutrition outcomes is often mediated by geography (e.g., rurality and proximity to markets) (Kissoly et al., 2018; Koppmair et al., 2017), it is important to understand the potential for crop diversification to support communities through both subsistence and market activities (Tobin et al., 2019). In other words, not all small-scale farm households must diversify their production to enjoy the benefits of the wider availability of diverse crops in their communities (Remans et al., 2015).

Given the diversity of mechanisms that may link crop diversity to food and nutritional outcomes, and the multiple levels at which they may operate, systematic attention to differences in effects across spatial scales is needed. This paper, therefore, asks: at what scale, if any, does crop diversity offer benefits to food and nutrition security among small-scale farm households? As Koppmair et al. (2017: 325) stress in their study of dietary diversity, market access, and production, “appropriate levels of diversification are a question of scale.” That is, guidance on whether a household should diversify should depend on its context and its relative ability to access nutritious food locally through other means (Remans et al., 2015). Questions of access to nutritious food must look beyond the household level to consider the availability and quality of food being produced locally or regionally, whether by local markets or other forms of exchange (Tobin et al., 2019). We address this gap by examining the association between crop diversification and children’s dietary diversity, measuring crop production from the village to regional scales across 10 SSA countries. Importantly, we also account for the importance of context by extending our analyses to account for potential cross-national variation in the relationship between crop and dietary diversity. Together, these results yield important insights into the spatial dimensions of the nutritional benefits to crop diversification.

## 3 Materials and methods

### 3.1 Data

We combine and analyze data from the DHS Program and high-resolution agricultural production estimates. The DHS Program is our source for nutrition data and information on key covariates included in the regression analyses. The DHS Program has conducted nationally representative cross-sectional surveys across a large number of low- and middle-income countries globally. The core questionnaire is standardized across countries to allow for comparisons across space and over time. Extracting data from IPUMS-DHS (Boyle et al., 2020), we use all surveys from SSA that include geo-coordinates of DHS clusters (i.e., communities) that were implemented between 1995 and 2005 – within 5 years of the circa-2000 agricultural data used to measure crop diversity^2^, and that have sufficient data on food group intake to facilitate our nutritional analyses. We assume that the structure of agricultural production does not change significantly during this window of a few years for which production data is available. A total of 11 samples from 10 countries meet these criteria and were used in the analyses presented in this paper. Observations for which key variables were not available were also dropped, and the universe for our focal outcome variable was limited to the youngest child of each mother within a household. We also exclude children under 6 months of age, considering the minimal dietary importance of food crops for breastfeeding infants.^3^ After these restrictions, the total sample for the main analysis is 43,477 children aged between 6–59 months. These children were sampled from 22,437 households (more than one mother can reside within a household), resulting in an average of ~2 children per household.

All crop diversity data were obtained from the agricultural production estimates produced by Monfreda et al. (2008). These estimates are derived from remotely sensed land cover data and agricultural censuses and surveys around the world (collected primarily by the Food and Agriculture Organization of the United Nations (FAO)). The resulting dataset includes gridded estimates of harvested area and yield for 175 crops globally circa 2000, at approximately 10-km^2^ resolution. We measured crop diversity by calculating the crop production figures provided by Monfreda et al. as a proportion of the total cropland extent (area) (i.e., the area allocated to particular crop species) for each of the focal spatial units in the study (e.g., 10-, 25-, and 50-km radii around the DHS household cluster)^4^.

### 3.2 Dependent variable

We extracted DHS data on key nutrition-related indicators as available across our 10 countries of interest (Table 1). The main dietary variable of interest is the Household Dietary Diversity Score (HDDS-7, referred to as HDDS throughout this paper), which we measure as a count of the consumption of seven food groups (starches and tubers, legumes, dairy, animal protein, vitamin A-rich vegetables and fruits, other vegetables and fruits, and fats and oils) over a 24-h recall period for the youngest child of each mother in the household, as reported by that child’s mother.^5^ 24-h recall periods are commonly used across the literature on children’s food security (Zeinalabedini et al., 2023) and, in comparison to 7-day recall periods, have generally lower respondent bias. That said, 24-h recalls may unintentionally not account for some food groups that may be consumed regularly within a household, but not daily (Manikas et al., 2023). Dietary diversity goes a step past simple food security measurements by considering the nutrient adequacy of different foods and food groups (Ruel, 2003). HDDS is considered to be a good indicator of broader nutritional status, dietary adequacy, and overall food security (Herforth & Ballard, 2016; Sibhatu et al., 2015). Above 6 months, the WHO and UNICEF (2017) defines a minimum acceptable diet as one that includes four or more of the seven food groups we account for above, including breast milk for those children under 23 months. We follow Tobin et al. (2019) in using HDDS-7 since data for more comprehensive HDDS measures are not available. F

### 3.3 Independent variables

To understand the association between crop diversity and children’s dietary diversity, we extracted agricultural production estimates produced by Monfreda et al. (2008). We started with the full set of 175 crop species, which was reduced to 112 species that can be found within SSA. With these data, we calculate the proportion of area dedicated to each crop species (following Mayala et al., 2018) within buffers of 10-km, 25-km, and 50-km around DHS cluster locations^6^, and then calculate the Simpson’s Diversity Index (SDI). SDI takes into consideration both crop species richness (i.e., the total number of species present) and evenness (i.e., the relative abundance of a species compared to the total number of species found within an area) (Jones et al., 2014). Richness is accounted for in SDI and other diversity indices simply based on the number of species present. Unlike some other diversity indices, the SDI calculation is also inclusive to evenness (DeJong, 1975), so that, for example, a field with a high number of species but that is mainly dedicated to one or a couple of staple crops would still have a relatively low SDI. SDI can be interpreted whereby 1 means infinite diversity and 0 means zero diversity (i.e., a monoculture). Within our models, we also control for several other individual contextual variables (Table 1), including mother’s age and education, and child’s sex and age, both of which have been found to influence children’s nutritional outcomes (Ruel et al., 2018). Finally, we include variables for total area devoted to cropland (i.e., proportion of all land within a given buffer devoted to growing crops) and pastureland (i.e., proportion of all land within a given buffer devoted to animal grazing), and precipitation change (difference in millimeters between 2000 and 2005^7^) within a 10-km buffer around each DHS cluster location. These variables were extracted from the DHS Geospatial Covariates dataset (Mayala & Donohue, 2022).

### 3.4 Analyses

We fit a series of Poisson regression models to evaluate the relationship between crop diversity at different scales and children’s dietary diversity, which take the following general form:

$$\begin{array}{l} D_{i\left(s\right)}=Poisson({\hat{D}}_{i\left(s\right)})\;with\;\log({\hat{D}}_{i\left(s\right)})\\ =a_{r}+\delta SDI_{c}+\beta X_{i\left(s\right)}+\lambda {\text{P}}_{c\left(s\right)} \end{array}$$

All models assume that dietary diversity (D) for child (i), drawn from sample (s), is a function of SDI for location (c) (corresponding to the shared geocoordinates of households in a DHS cluster), which is measured across an area defined by buffers of 10-km, 25-km, or 50-km around that location. The models also include a set of individual-level control variables X, including child sex and age, maternal educational attainment and age, and sex of household head; and a set of clusterlevel place characteristics (P), including rural (urban) status of enumeration area, travel time to nearest city of 50,000 or more population as a measure of remoteness, the respective shares of land within a 10-km radius used for cropland and pastureland measured circa 2000 (matching the timing of the DHS surveys), and precipitation change (2000 to 2005). We include individual- and place-level characteristics that are often correlated with crop and/or dietary diversity and could therefore confound the focal associations (see Jones et al., 2014 and Sibhatu et al., 2015). We also include sample-specific region fixed effects (α_r_) to account for systematic sample-specific variation between regions (i.e., first subnational administrative units) in local crop diversity, children’s dietary diversity, and other unobserved variables. To account for potential variation in the association between crop diversity and dietary diversity across our geographically large sample, we also fit a series of similarly-specified Poisson models for each country separately. This approach allows for more nuanced understanding of how the relationship between HDDS and crop diversity may differ depending on country context.

## 4 Results

**Fig. 1:**
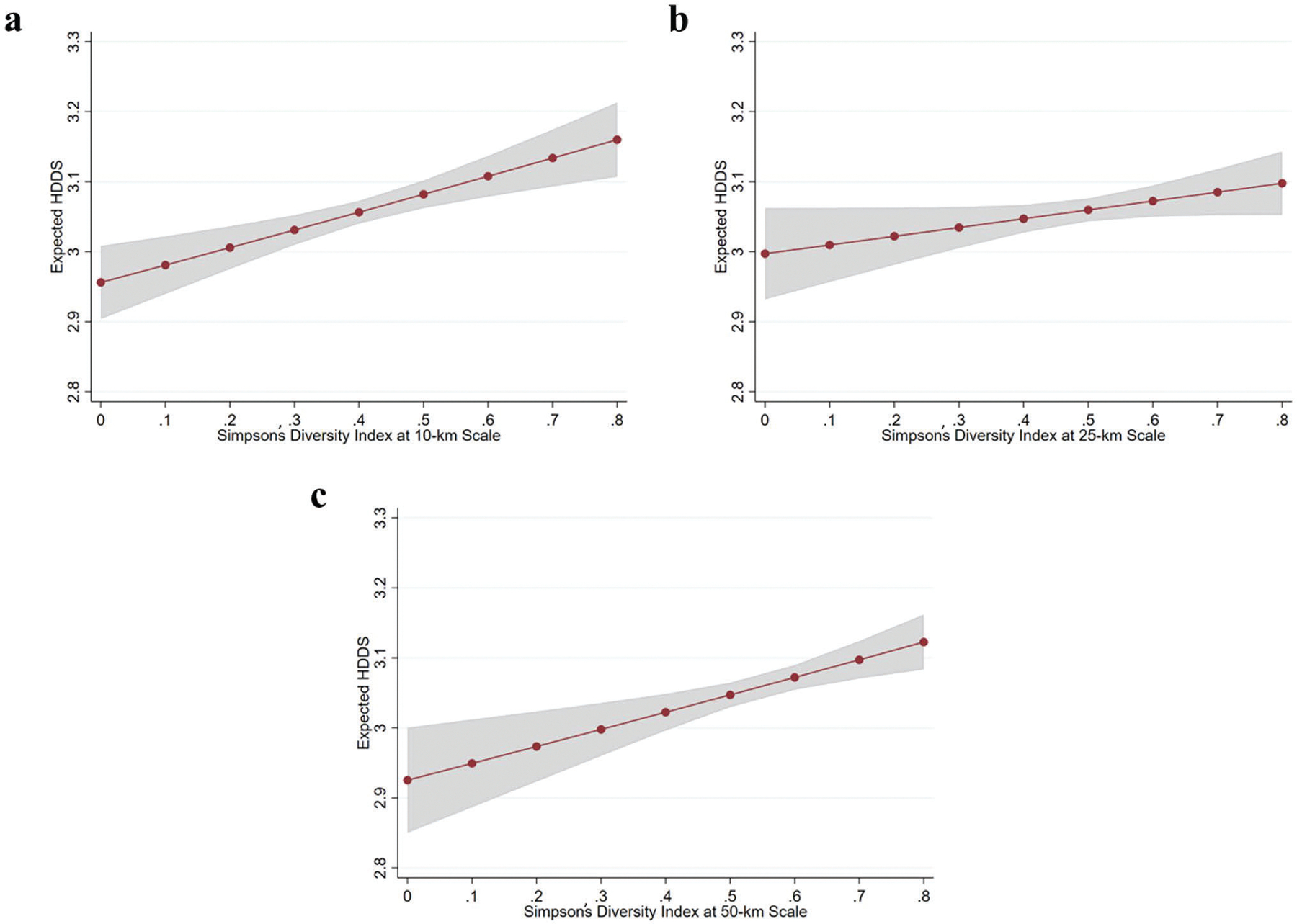
Predicted HDDS by SDI, derived from naïve bivariate poisson regression models. Shaded gray lines represent 95% confidence interval

First, we conduct three naïve bivariate regressions to understand the relationship between crop diversity, at our three focal scales of analysis, and children’s dietary diversity across our entire 10-country sample. The first model measures the association between SDI and dietary diversity when SDI is measured within a 10-km buffer and reveals a significant and positive association. The regression coefficients represent the difference between minimum- and maximum-diversity settings given how SDI is operationalized (i.e., ranging from 0 to 1), which is practically implausible given the observed variation in SDI across the sample. We, therefore, interpret the results by deriving predicted dietary diversity scores from the models (holding covariates at their means when included in the model), which can provide insight into the expected differences in dietary diversity along a more realistic continuum of SDI values.^8^ As shown in Fig. 1a, an increase in SDI (10-km) from 0.3 to 0.7 – a range that corresponds to approximately ± 0.2 (or roughly 2 standard deviations) around the mean SDI across scales – implies a 0.10-point increase in the expected dietary diversity score. Figures 1b and c examine this relationship when SDI is measured within 25- and 50-km buffers. These associations are also statistically significant, and of a similar magnitude to the estimates at the 10-km scale. A change in SDI from 0.3 to 0.7 corresponds to 0.05- and 0.1-point increases in expected dietary diversity scores at the 25- and 50-km scales, respectively.

We then account for potential confounding variables by fitting a series of regression models that include control variables (Table 2). After adjusting for sociodemographic and geographic factors, we find that crop diversity is negatively associated with HDDS at 10-km (M1) and 25-km (M2) scales. This negative association between SDI and child dietary diversity runs contrary to the positive association observed in the naïve bivariate models. The association between SDI at the 50-km scale and dietary diversity (M3) is not statistically significant. The change in the direction of the relationship between SDI and child dietary diversity with the addition of control variables emphasizes that crop diversity (as measured by SDI) is one among many significant predictors of dietary diversity, and the relationships between crop diversity, social and environmental context, and child dietary diversity are more complex than what is accounted for in the naïve models (Fig. 1).

**Fig. 2:**
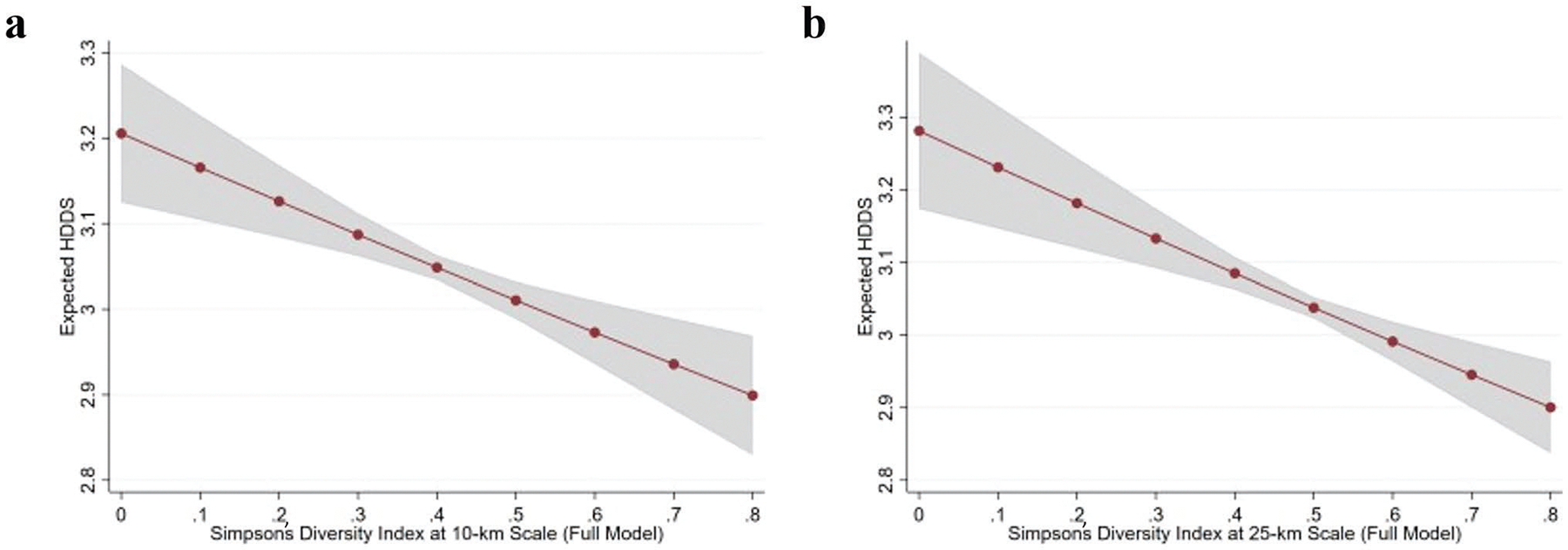
Predicted HDDS by SDI, derived from multivariate Poisson regression models. Shaded gray lines represent 95% confidence interval

To better understand the magnitude of the association between SDI and child dietary diversity, we again plot the predicted dietary diversity scores across a range of SDI values while holding all other variables at their means (Fig. 2). When crop diversity is measured at the 10-km scale (Fig. 2a), a change in SDI from 0.3 to 0.7 implies a 0.15-point decrease in the expected dietary diversity score. At the 25-km level (Fig. 2b), a change in SDI from 0.3 to 0.7 implies a 0.19-point decrease in the expected dietary diversity score. These results support two straightforward conclusions. First, the decision to measure crop diversity at the 10-km or 25-km scale across our pooled sample does not have substantively meaningful impacts on our conclusions about the link between SDI and child dietary diversity. Second, the magnitude of estimated effects is modest. The marginal changes in predicted HDDS reported above represent only small fractions of the sample wide mean HDDS of 3.1 (SD = 1.5).

**Fig. 3:**
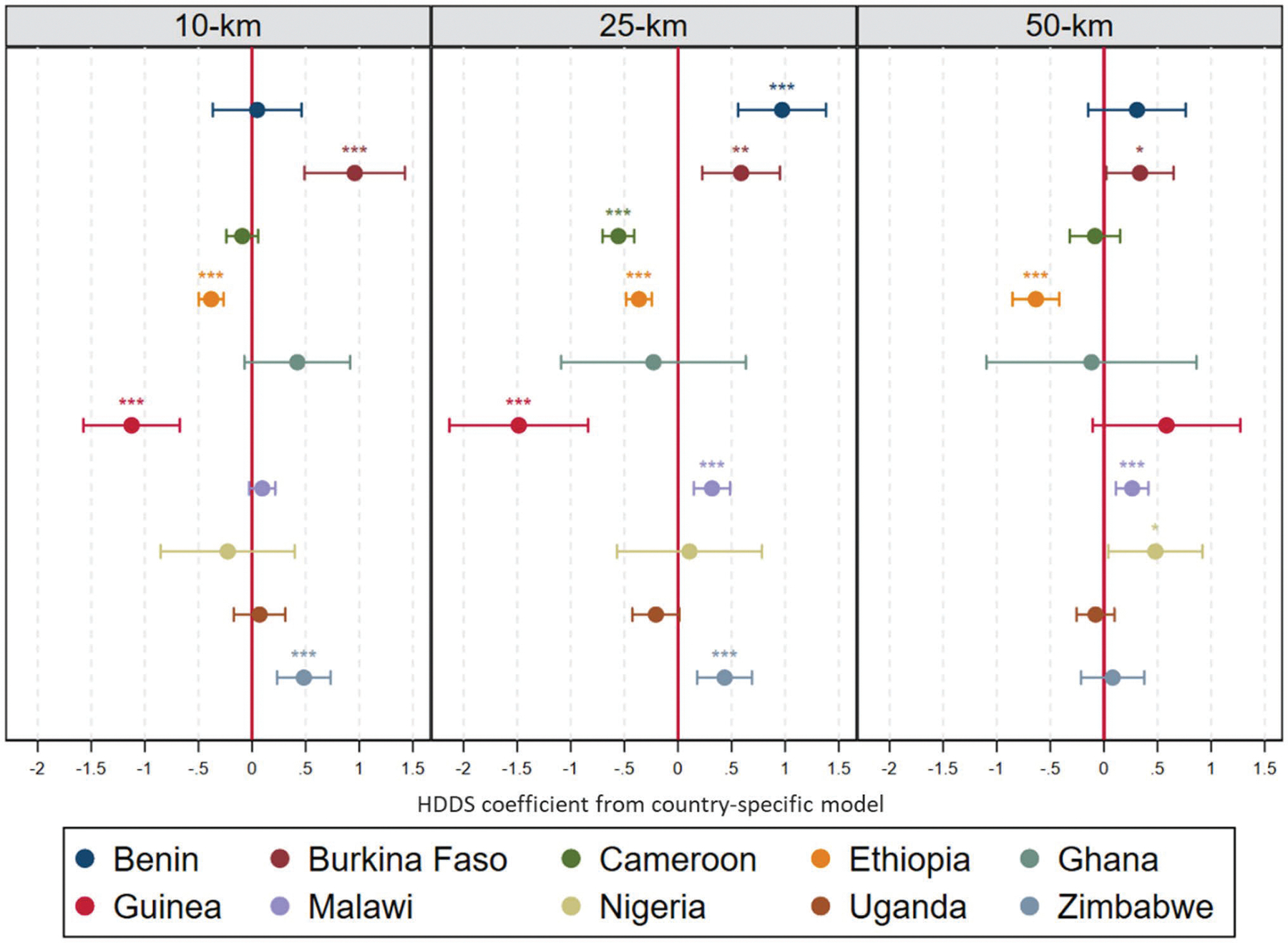
Summary of poisson regression models of HDDS at different scales by country. Lines from point estimate represent 95% confidence interval, * = p < 0.05, ** = p < 0.01, *** = p < 0.001

Based on the findings in Table 2, which underscore the significance of a wide range of control and contextual variables in influencing the statistical relationship between SDI and HDDS, the final set of analyses account for potentially important contextual differences between the 10 SSA countries in our sample. We do so by fitting individual, fully controlled models for each country and at each of the three focal scales. Figure 3 summarizes the direction and significance of the regression coefficients for the association between crop diversity and dietary diversity by country and scale (full results included in the Supplemental Material, Tables B). The results reveal a high degree of variability in the relationship between crop diversity and dietary diversity across countries. For instance, when we measure SDI at a 10-km scale, crop diversity is positively associated with dietary diversity among children in only two countries – Burkina Faso and Zimbabwe. In contrast, the association is negative in Ethiopia and Guinea, and there are no significant associations in the remaining counties. When SDI is measured at the 25-km scale, crop diversity is positively associated with dietary diversity in four countries (Benin, Burkina Faso, Malawi, and Zimbabwe), and negatively associated with dietary diversity in three countries (Cameroon, Ethiopia, and Guinea). There is no significant association between SDI at the 25-km scale and dietary diversity in Nigeria, Ghana, or Uganda. Finally, in the last set of models – when SDI is measured at the 50-km scale – we detect a positive association between crop diversity and dietary diversity within three countries (Burkina Faso, Guinea, and Nigeria) but a negative association in one country (Ethiopia).

Despite the variation in associations across countries, we note that the magnitude of effects for each of our sample-specific analyses is markedly larger than in our pooled analyses, as indicated by the size of coefficients in Fig. 3. To provide further insight, we looked at the marginal effect of increasing SDI from 0.3 to 0.7 – which, again, corresponds to roughly 2 standard deviations about the sample mean of 0.5 – on HDDS across all country samples and scales for which significance was obtained. When measuring SDI at the 10-km scale, the effects are: + 1.13 (Burkina Faso), + 0.70 (Zimbabwe), −0.42 (Ethiopia), and −1.13 (Guinea). When we measure SDI at the 25-km scale the effects are: + 1.25 (Benin), + 0.63 (Burkina Faso), + 0.61 (Zimbabwe), + 0.37 (Malawi), −0.42 (Ethiopia), −0.85 (Cameroon), and −1.58 (Guinea). Finally, when we measure SDI at the 50-km scale the effects are: + 0.35 (Burkina Faso), + 0.30 (Malawi), + 0.67 (Nigeria), and −0.79 (Ethiopia). These effects are, in general, substantially larger than those shown in Figs. 1 and 2, which highlights that SDI has a more substantively meaningful impact on HDDS (either positively or negatively) when analyzing the association within each country and by controlling for other important contextual factors. Moreover, it is interesting to note that the effect size of SDI on HDDS does not seem to increase systematically as the focal scale increases – even though SDI does increase as the buffer zone is widened across countries and samples (Tables 1 and A5).

Lastly, we also note that the differences across scales within countries are fairly modest. In all cases, the direction of the coefficient remains consistent across scales or the coefficient switches from statistically significant in one direction at one scale to not statistically significant at another scale. The implication is that inferences about crop diversity’s effects are more heterogeneous across countries than across focal scales within countries.

## 5 Discussion

This study contributes to the expanding literature on crop diversification and food and nutrition security by investigating how local and regional crop diversity is associated with children’s dietary diversity in 10 SSA countries. This study builds on recent research that suggests that the effects of crop diversification on health and nutrition outcomes may be context- and/or scale-specific and helps address a gap in the empirical evidence on these issues (Khonje et al., 2022; Tobin et al., 2019). Here, we place new emphasis on the spatial heterogeneity in these associations by measuring crop diversity across multiple scales – from the village to regional levels – and across multiple countries.

Our results point to at least three major conclusions. First, crop diversity is negatively associated with children’s dietary diversity across our entire 10-country sample, at least when crop production is measured within relatively short distances of the child’s community of residence (i.e., 10- and 25-km buffers). This inverse relationship is somewhat unexpected. Although prior studies (which have largely been conducted at the household level) have found inconsistent associations between crop diversity and food and nutritional security, estimates have tended to yield either positive or non-significant associations, not negative ones (Jones, 2017a; Kumar et al., 2018; Muthini et al., 2020). That being said, while these negative associations are statistically significant, their coefficients are generally small – with limited substantive significance, at most. Indeed, the small effect sizes of our pooled analyses are consistent with previous studies that have looked at the association between production diversity and nutrition (Khonje et al., 2022; Sibhatu & Qaim, 2018). This negative association is nonetheless notable and merits further attention, as it suggests the link between crop diversification and children’s dietary diversity is not as clear-cut as sometimes portrayed.

There are a multiplicity of reasons that could cause this negative association. For instance, it is not difficult to imagine a situation in which increasing crop diversity may be more beneficial in some areas over others, dependent on the existing supply and demand of certain crops within that area (e.g., diversifying more fields to vegetables in areas where there is already a high (over)supply of vegetables may not support diversified livelihoods but instead cause market oversaturation). For interventions seeking to enhance diversification, this point foregrounds the importance of understanding exactly which or what types of crops may lead to income growth (Jones, 2017a, b). For example, more diversity into non-high-value crops may not help households, especially if the goal is to increase nutrition outcomes through the income pathway. Moreover, crop diversification may not have much of an effect on dietary diversity at all through the consumption pathway if the food groups which are lacking in a child’s diet are non-crop related (i.e., dairy or animal protein food groups). The latter tend to be the first food groups sacrificed during lean periods (Fraval et al., 2019), especially as their sale often provides a buffer for households after climate-related crop losses (Bakhtsiyarava & Grace, 2021). Importantly, the patterns shown here do not mean that crop diversification in all cases leads to negative outcomes but supports the widely held understanding that not all households may benefit from diversification, especially given the importance of context (e.g., extension services, access to markets, local availability of fresh foods, etc.) in determining the efficacy of diversification for smallholder livelihoods (Remans et al., 2015).^9^

Second, we find that the overall association between crop diversity and children’s dietary diversity masks a high degree of heterogeneity between countries, which is consistent with a recent review of findings on this topic from across the world (Nandi et al., 2021). Indeed, country-stratified models show that the direction and magnitude of effects vary considerably across the 10 countries in our sample (further summarized in Table 3). For example, while coefficients of local crop diversity at the 10- and/or 25-km scales are consistently statistically significant and negative in 3 of the 10 countries in our sample (Cameroon, Ethiopia, and Guinea), in other countries coefficients shift to positive or non-significant. Indeed, the results of the overall and country-specific models diverge in most of the countries within our sample, although we do detect consistently positive associations in countries such as Burkina Faso and Zimbabwe. The clear implication is that the spatial processes linking local crop diversity and dietary diversity are context-specific and therefore merit study with localized models, which may be a useful complement or substitute to the pooled cross-national studies that are increasingly common in food and nutritional security literature (Anttila-Hughes et al., 2021; Cooper et al., 2019; Thiede & Strube, 2020). As such, while this paper does not specifically answer why the association between HDDS and SDI may vary across countries, we urge future studies to investigate how crop diversification may relate to children’s dietary intake as mediated by other social, environmental, and political factors that are often highly specific to countries and sub-regions.

Third, the scale at which crop diversity is measured has generally modest but non-trivial implications for inferences about its association with dietary diversity. In four countries – Burkina Faso, Ethiopia, Ghana, and Uganda – the relationship (or lack thereof) between crop diversity and dietary diversity is consistent across all three levels of measurement. These relationships vary in the other six countries within the sample. However, in all six countries, the relationship changes from statistically significant to non-significant across scales but does not switch from statistically significant in one direction to statistically significant in another. As we suggest below, these differences underscore the importance of understanding what structural factors might drive variability across space.

Our findings raise many questions as to why the presence of crop diversity may be more beneficial at some scales and in some contexts than in others. To provide a few examples, Table 3 shows that, in Burkina Faso, crop diversity is positively associated with HDDS regardless of scale. However, Fig. 3 provides further detail that the average coefficient of HDDS decreases as the scale gets larger, implying that crop diversification has a more positive effect on dietary diversity at village than regional scales. This is confirmed when looking at the marginal effects of a change in SDI from 0.3 to 0.7 on HDDS, which decrease as the scale of analysis becomes larger (10-km: + 1.13, 25-km: + 0.63, 50 km: + 0.35), despite the slight mechanical increase of SDI as the scale of analysis gets larger (Table A5). In contrast, analyses for Malawi are only significant at the 25-km and 50-km scales. There, the marginal effects are similar for both scales, suggesting that having crop diversity nearby (but not directly in one’s community) may be sufficient to enhance children’s dietary diversity. These findings underscore the fact that the structure, roles, and capacities of markets and other forms of exchange vary substantially across countries. For example, a statistically significant positive association detected at the 10-km scale could indicate that local markets and intra-village exchanges matter most in enhancing nutrition outcomes, while a similarly positive coefficient at the 50-km scale could indicate that regional markets and extra-village exchanges may be more important.

While our analyses ultimately cannot explain why exactly these results occurred, possible explanations are numerous and include the relative importance of market sales of food crops in different regions and countries, the accessibility and quality of transportation infrastructure, and cultural norms around foods most likely to be consumed. As Nandi et al. (2021) suggest in their study, special attention should thus be paid not solely to the existence of crop diversity as a pathway to positive dietary and nutrition outcomes, but also to other factors such as seasonality, micronutrient availability, access to transportation to get to markets, and the quality of local markets in moderating these relationships. Referring to our findings, this may be why crop diversity may be positively associated with dietary diversity at some scales, but not in others.

## 6 Conclusions and directions for future research

Both the results and limitations inherent to our study raise several questions for further research. First, our results reveal variation in the association between crop diversity and dietary diversity across scales, but do not definitively identify the substantive processes that explain these differences. Given that dietary diversity can reduce chronic food insecurity (Bakhtsiyarava & Grace, 2021), lead to reductions in the prevalence of stunting (Akombi et al., 2017), and also reduce childhood mortality and increase the quality of children’s lives (Afshin et al., 2019; Willett et al., 2019), crop diversification remains an important strategy to improving the health and nutritional status of small-scale farming households in many places (Jones et al., 2017a). That being said, special attention to local context is necessary when developing interventions, as is the need to separate out and concentrate on different pathways to increasing dietary diversity (i.e., through direct/local consumption or income generation). As exemplified by the variation in our analyses, more work – likely through primary and mixed-methods data collection – is needed to identify the social, economic, and ecological processes that influence how local crop diversification translates into children’s diets.

Secondly, this study uses distance-based measures (10-, 25-, and 50-km buffers) of local crop diversity instead of, for example, the various administrative units that characterize the countries in our sample. We do so given cross-national differences in the size and substantive meaning of those administrative units across countries. However, such distance-based measures do not account for political, social, and ecological barriers that may shape how crop diversity and its exchange within a given radius may influence children’s nutritional outcomes. Indeed, the extent to which a given area corresponds to a marketshed in which agricultural commodities are exchanged may vary considerably (Nandi et al., 2021). Future research should build on our findings to consider other ways to measure local crop diversity across the scales and spaces that are most relevant to the households that reside within them.

Third, the DHS samples we use did not collect as rich of dietary diversity data as other sources (e.g., more recent HDDS-12 scales) and did not collect household crop production data (e.g., as done in the Living Standards Measurement Survey-Integrated Surveys on Agriculture (LSMS-ISA) effort led by the World Bank), which would enable comparisons of effects between household and local crop diversification. While our research addresses a gap in knowledge about how local and regional crop diversity affects individual-level food consumption, future research that incorporates production data both for a household and its locality would facilitate further understanding of the multi-scalar relationship between crop and dietary diversity. Relatedly, analyses that compare the effects of crop diversification on dietary diversity for different members of the same household (as explored in Singh et al., 2020) would add further depth to our understanding of how the nutritional impacts of crop diversity are distributed within families.

Fourth and finally, our study focuses on only 10 countries in SSA, using data from between 1995 and 2005. These restrictions were necessary to appropriately link the DHS and crop production datasets but nonetheless represent significant limitations given changes in agriculture and nutrition over recent years and, as our results demonstrate, the high degree of spatial heterogeneity in the relationship between crop and dietary diversity. Future research should therefore draw on and/or collect additional data to examine these questions in other countries and regions around the world. Likewise, future work should examine the important differences in sub-populations that were not explored in this paper (e.g., women, men, older youth, displaced populations). Despite these limitations, however, we expect that our study – especially its new focus on spatial heterogeneity – will serve as a foundation for addressing these important questions about the links between crop diversification and child nutrition. Moreover, while in many respects our study prompted more questions than answers, we hope that the variability revealed through our analysis foregrounds the continued importance of studying crop diversification as one among many methods of enhancing the health and livelihoods of small-scale farming households.

## Supplementary Material

supplement

**Supplementary Information** The online version contains supplementary material available at https://doi.org/10.1007/s12571-024-01458-9.

## Figures and Tables

**Table 1:** Weighted descriptive statistics (n = 43,477)

Variable	Mean^a^	Std. dev	Min	Max

Simpson's Diversity Index, 10-km buffer	0.40	0.13	0.00	0.79
Simpson's Diversity Index, 25-km buffer	0.48	0.12	0.00	0.81
Simpson's Diversity Index, 50-km buffer	0.54	0.11	0.00	0.81
HDDS	3.06	1.45	1.00	7.00
Sex of child = female	0.50	-	0.00	1.00
Head of household = female	0.16	-	0.00	1.00
Age of mother (years)	28.68	7.20	15.00	49.00
Age of child				
6 months—11 months	0.17	-	0.00	1.00
12 months—23 months	0.37	-	0.00	1.00
24 months—35 months	0.29	-	0.00	1.00
36 months—47 months	0.09	-	0.00	1.00
48 months—59 months	0.08	-	0.00	1.00
Education of mother = primary school +	0.47	-	0.00	1.00
Rural = yes	0.80	-	0.00	1.00
Remoteness^b^				
Q1	0.18	-	0.00	1.00
Q2	0.20	-	0.00	1.00
Q3	0.19	-	0.00	1.00
Q4	0.21	-	0.00	1.00
Q5	0.22	-	0.00	1.00
Cropland, 10 km radius	0.20	0.18	0.00	1.00
Pastureland, 10 km radius	0.14	0.16	0.00	0.96
Annual precipitation change in mm (2000–2005^c^)	91.52	32.65	16.66	205.50
Sample				
Cameroon 2004	0.07	-	0.00	1.00
Benin 2001	0.05	-	0.00	1.00
Burkina Faso 2003	0.12	-	0.00	1.00
Ethiopia 2000	0.23	-	0.00	1.00
Ghana 2003	0.04	-	0.00	1.00
Guinea 2005	0.06	-	0.00	1.00
Malawi 2000	0.13	-	0.00	1.00
Malawi 2004	0.12		0.00	1.00
Nigeria 2003	0.05	-	0.00	1.00
Zimbabwe 1999	0.07	-	0.00	1.00
Uganda 2001	0.06	-	0.00	1.00

a Means of categorical variables can be interpreted as the proportion of the total sample that falls into that category

b Remoteness is defined as the approximate travel time to market a DHS point is to a population center of 50,000 or more people. For the purposes of analyses, this was broken down into quintiles where Q1 refers to “least remote” and Q5 refers to “most remote.”

c Precipitation change is calculated as the average annual rainfall in 2000 plus the average annual rainfall in 2005 divided by 2, measured in millimeters

**Table 2:** Poisson regressions of HDDS and Simpson’s diversity index at different scales

Variable	SDI M1: 10-km		SDI M2: 25-km		SDI M3: 50-km	
	Coef.	RSE	Coef.	RSE	Coef.	RSE

Simpson’s Diversity Index (SDI)	−0.126***	0.031	−0.154***	0.034	−0.077	0.041
Sex of child = female	0.013*	0.005	0.013**	0.005	0.013**	0.005
Head of household = female	−0.038***	0.006	−0.037***	0.006	−0.038***	0.006
Age of mother	0.001*	0.000	0.001*	0.000	0.001*	0.000
Age of child (ref = 6 months – 11 months)						
12 months—23 months	0.251***	0.008	0.251***	0.008	0.251***	0.008
24 months—35 months	0.332***	0.008	0.332***	0.008	0.332***	0.008
36 months—47 months	0.331***	0.012	0.331***	0.012	0.331***	0.012
48 months—59 months	0.319***	0.013	0.318***	0.013	0.319***	0.013
Education of mother = primary school +	0.137***	0.007	0.133***	0.007	0.133***	0.007
Rural = yes	−0.158***	0.009	−0.159***	0.009	−0.157***	0.009
Remoteness (ref = Q1)						
Q2	−0.012	0.010	−0.010	0.010	−0.011	0.010
Q3	−0.006	0.011	−0.002	0.011	−0.004	0.011
Q4	−0.019	0.011	−0.016	0.011	−0.018	0.011
Q5	−0.052***	0.012	−0.047***	0.012	−0.050***	0.012
Cropland, 10 km radius	−0.038*	0.020	−0.054**	0.018	−0.071***	0.017
Pastureland, 10 km radius	−0.093***	0.019	−0.091***	0.019	−0.101***	0.020
Annual Precipitation Change	0.002***	0.000	0.002***	0.000	0.002***	0.000
Sample Region Fixed Effects	Yes		Yes		Yes	
Constant	0.993***	0.042	0.891***	0.045	0.987***	0.047
Observations	41,685		41,685		41,685	
Wald χ^2^(102)	13258.900		13281.230		13238.740	
Prob > χ^2^	0.000***		0.000***		0.000**	

*RSE* robust standard error

* p < 0.01;

** p < 0.05;

*** p < 0.001

**Table 3:** Summary of estimated relationships between SDI and HDDS across spatial scales

Sample	Scale of Buffer		
	10-km	25-km	50-km

Benin	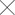	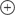	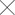
Burkina Faso	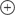	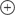	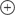
Cameroon	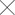	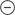	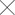
Ethiopia	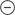	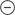	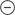
Ghana	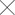	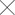	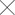
Guinea	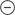	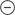	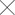
Malawi	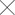	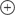	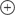
Nigeria	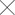	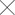	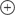
Uganda	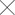	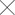	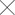
Zimbabwe	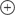	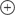	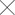
All Countries	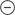	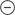	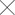

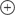
 = positive association (p < 0.05), 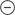 = negative association (p < 0.05), 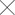 = no association

## Footnotes

1 According to some estimates, rural households account for up to 90% of total households in poverty across SSA (OECD/FAO, 2016).

2 DHS geocoordinates are displaced by up to 10-km to protect respondent confidentiality. The minimum buffer we use to measure local crop diversity is also 10-km, ensuring that each of our buffers captures the true location of DHS communities.

3 As breastfeeding can be an important source of nutrients for children into their toddler years (Ogbo et al., 2017), we also ran our main models using a sample limited to only children between the ages of 24–59 months, which can be found in the Supplemental Material, Tables A.

4 Monfreda et al.’s (2008) data have been used widely across similar studies, especially those seeking to understand changes in land use and their association with food availability and cropping choices over time (Barbieri et al., 2019; Nicholson et al., 2021; Renard & Tilman, 2019; Zhao et al., 2017). We also note that other scholars have recently produced more updated measures of crop diversity (e.g., Nicholson et al., 2021; IFPRI, 2020) using similar original source data, but many lack the same spatial resolution, geographic coverage, and breadth of crop species needed for this study and as produced by Monfreda et al. (2008). Our use of Monfreda et al. (2008) also permits direct comparisons with Tobin et al. (2019), upon which this study builds.

5 The limited universe for the dietary diversity data included in the DHS prevents us from examining the potentially important intrahousehold inequalities in dietary diversity that have been found in other contexts (e.g., Singh et al., 2020).

6 Despite previous research which has shown the positive impact that food-specific SDI (e.g., SDIs calculated only with crops that can be eaten for food) can have on health outcomes, we include all 112 crops available for our analyses. This is to ensure that crop diversity grown for a multiplicity of purposes beyond direct subsistence is accounted for, such as the indirect benefits that market sales can have on consumption.

7 Change in precipitation is a proxy for experience of drought, which has been shown to impact childhood nutritional outcomes (Cooper et al., 2019). Year 2000 is the earliest for which data is available.

8 For reference, summaries of SDI values for all samples and scales can be found in Table A5 of the Supplemental Material.

9 Due to the nature of our initial quantitative analyses which pool data across 10 different countries, we are inherently limited in unraveling the exact reasons that crop diversity may have negatively impacted HDDS at smaller scales of analysis and, at the same time, risk bias from omitted variables or of the results being driven by processes in a sub-set of countries. For example, it appears that the negative effect within our pooled analysis is largely explained by Ethiopia – a country which 20 years ago (the time period we have data for) was extremely fragmented and fraught with extreme degrees of poverty. Moreover, it must also be acknowledged that measurements of SDI are imperfect – for instance, even if SDI is relatively high, what matters is where that diversity actually ends up, meaning that if large (and diverse) landowners are driving SDI estimates but are producing crops for extra-local consumers, this can affect HDDS minimally or even negatively.
