# Supplementary material for: The association between crop diversity and children’s dietary diversity: multi-scalar and cross-national comparisons
Source: Food Secur. Author manuscript; Available in PMC 2026 May 28. (PMC13215701; doi:10.1007/s12571-024-01458-9)
Supplement: supplement [file NIHMS2168716-supplement-supplement.docx]

**Tables A**

**Table A1. Poisson Regressions of HHDS and Simpson’s Diversity Index at Different Scales**

| **Variable** | **SDI M1: 10-km** | | **SDI M2: 25-km** | | | **SDI M3: 50-km** | | |
| --- | --- | --- | --- | --- | --- | --- | --- | --- |
|  | **Coef.** | **RSE** | | **Coef.** | **RSE** | | **Coef.** | **RSE** |
| Simpson's Diversity Index (SDI) | -0.180*** | 0.043 | | -0.207*** | 0.047 | | -0.176** | 0.061 |
| Sex of child = female | 0.013* | 0.007 | | 0.012 | 0.007 | | 0.013 | 0.007 |
| Head of household = female | -0.015 | 0.008 | | -0.015 | 0.009 | | -0.015 | 0.009 |
| Age of mother | 0.001 | 0.000 | | 0.001 | 0.000 | | 0.001 | 0.000 |
| Age of child (ref 24-35 months) |  |  | |  |  | |  |  |
| 36 months - 47 months | 0.008 | 0.011 | | 0.008 | 0.011 | | 0.008 | 0.012 |
| 48 months - 59 months | -0.001 | 0.012 | | 0.001 | 0.012 | | -0.001 | 0.013 |
| Education of mother = primary school + | 0.139*** | 0.009 | | 0.139*** | 0.009 | | 0.139*** | 0.009 |
| Rural = yes | -0.182*** | 0.013 | | -0.183*** | 0.012 | | -0.182*** | 0.013 |
| Remoteness (ref = Q1) |  |  | |  |  | |  |  |
| Q2 | -0.009 | 0.014 | | -0.006 | 0.014 | | -0.006 | 0.014 |
| Q3 | -0.003 | 0.016 | | 0.004 | 0.016 | | -0.004 | 0.016 |
| Q4 | -0.001 | 0.016 | | 0.004 | 0.016 | | 0.003 | 0.016 |
| Q5 | -0.030 | 0.017 | | -0.034 | 0.017 | | -0.025 | 0.017 |
| Cropland, 10-km radius | -0.011 | 0.030 | | -0.036 | 0.028 | | -0.059* | 0.027 |
| Pastureland, 10-km radius | -0.076** | 0.027 | | -0.075** | 0.027 | | -0.082** | 0.027 |
| Annual Precipitation Change | 0.003*** | 0.000 | | 0.002*** | 0.000 | | 0.002*** | 0.000 |
| Sample Region Fixed Effects | Yes |  | | Yes |  | | Yes |  |
| Constant | 1.252*** | 0.058 | | 1.295*** | 0.062 | | 0.987*** | 0.047 |
| Observations | 19,284  7315.590  0.000*** | | 19,284  7321.750  0.000*** | | | 19,284  7298.290  0.000** | | |
| Wald χ ^2^(102) |  |  |  |  |  |  |  |  |
| Prob > χ^2^ |  |  |  |  |  |  |  |  |

**Notes:** * = p < 0.05, ** = p < 0.01, *** = p < 0.001, RSE = robust standard error

**Table A2. Poisson Regressions of HDDS and Simpson’s Diversity at 10-km Scale**

|  | **Country Coef. (RSE)** | | | | | | | | | |
| --- | --- | --- | --- | --- | --- | --- | --- | --- | --- | --- |
| Variable | Benin | Burkina Faso | Cameroon | Ethiopia | Ghana | Guinea | Malawi | Nigeria | Uganda | Zimbabwe |
| Simpson's Diversity | -0.334 | 0.660* | -0.049 | -0.350*** | -0.006 | -0.819 | 0.102 | -0.506 | -0.303 | 0.622*** |
|  | (0.304) | (0.295) | (0.144) | (0.070) | (0.329) | (0.639) | (0.092) | (0.495) | (0.212) | (0.147) |
| Sex of child = female | -0.019 | -0.052** | 0.080*** | 0.013 | -0.033 | 0.158*** | 0.005 | 0.057 | 0.048 | 0.031* |
|  | (0.024) | (0.020) | (0.023) | (0.014) | (0.034) | (0.033) | (0.014) | (0.033) | (0.028) | (0.015) |
| Head of household = female | -0.021 | 0.079* | -0.003 | -0.037 | 0.098* | -0.054 | -0.049** | 0.028 | -0.079* | 0.026 |
|  | (0.047) | (0.038) | (0.022) | (0.020) | (0.040) | (0.067) | (0.018) | (0.051) | (0.034) | (0.016) |
| Age of mother | 0.005** | 0.002 | -0.001 | -0.002 | 0.008*** | 0.006* | -0.002* | 0.002 | -0.001 | 0.002 |
|  | (0.002) | (0.001) | (0.002) | (0.001) | (0.002) | (0.003) | (0.001) | (0.002) | (0.002) | (0.001) |
| Age of child (ref = < 24 - 35 months) |  |  |  |  |  |  |  |  |  |  |
| 36 months - 47 months |  | -0.012 |  | 0.019 | -0.036 |  |  |  |  | -0.010 |
|  |  | (0.022) |  | (0.016) | (0.085) |  |  |  |  | (0.018) |
| 48 months - 59 months |  | 0.007 |  | -0.005 |  |  |  |  |  | 0.007 |
|  |  | (0.029) |  | (0.017) |  |  |  |  |  | (0.018) |
| Education of mother = primary school + | 0.026 | 0.171*** | -0.021 | 0.176*** | 0.133** | 0.106** | 0.170*** | 0.062 | 0.033 | 0.188*** |
|  | (0.031) | (0.031) | (0.041) | (0.019) | (0.042) | (0.038) | (0.017) | (0.036) | (0.039) | (0.028) |
| Rural = yes | -0.016 | -0.272*** | -0.045 | -0.166*** | 0.037 | -0.090 | -0.284*** | -0.110* | -0.228*** | -0.147** |
|  | (0.045) | (0.041) | (0.042) | (0.027) | (0.047) | (0.074) | (0.032) | (0.043) | (0.040) | (0.046) |
| Remoteness (ref = Q1) |  |  |  |  |  |  |  |  |  |  |
| Q2 | -0.014 | -0.018 | -0.012 | 0.037 | -0.105* | -0.484*** | 0.007 | 0.055 | 0.174*** | -0.074 |
|  | (0.054) | (0.043) | (0.046) | (0.037) | (0.051) | (0.070) | (0.034) | (0.054) | (0.042) | (0.046) |
| Q3 | 0.040 | 0.081 | 0.028 | -0.043 | -0.230** | -0.436*** | 0.049 | 0.040 | 0.153** | -0.106* |
|  | (0.062) | (0.046) | (0.050) | (0.039) | (0.072) | (0.070) | (0.036) | (0.064) | (0.052) | (0.049) |
| Q4 | -0.034 | 0.004 | -0.126* | -0.020 | -0.198** | -0.401*** | 0.071 | 0.210*** | 0.197*** | -0.129** |
|  | (0.067) | (0.051) | (0.058) | (0.038) | (0.069) | (0.076) | (0.037) | (0.062) | (0.053) | (0.049) |
| Q5 | 0.096 | 0.050 | -0.105 | -0.066 | -0.366*** | -0.463*** | 0.051 | 0.192* | 0.063 | -0.147** |
|  | (0.077) | (0.057) | (0.061) | (0.037) | (0.101) | (0.077) | (0.043) | (0.080) | (0.065) | (0.049) |
| Cropland, 10-km radius | -0.005 | -0.072 | -0.361** | 0.124 | -0.011 | 2.552*** | -0.127 | -0.193* | 0.061 | -0.002 |
|  | (0.131) | (0.153) | (0.128) | (0.076) | (0.128) | (0.492) | (0.081) | (0.083) | (0.071) | (0.160) |
| Pastureland, 10-km radius | -1.481** | -0.223 | -0.440* | -0.163 | -0.050 | -0.450** | -0.011 | 0.180 | -0.219* | -0.096* |
|  | (0.544) | (0.116) | (0.171) | (0.089) | (0.147) | (0.156) | (0.055) | (0.131) | (0.095) | (0.047) |
| Annual Precipitation Change | -0.006 | -0.006* | 0.005** | 0.004*** | 0.016*** | 0.004** | 0.001 | 0.000 | 0.001 | -0.003*** |
|  | (0.004) | (0.003) | (0.002) | (0.000) | (0.005) | (0.001) | (0.001) | (0.001) | (0.002) | (0.001) |
| Sample Region Fixed Effects | Yes | Yes | Yes | Yes | Yes | Yes | Yes | Yes | Yes | Yes |
| Constant | 2.000*** | 1.512*** | 1.248*** | 1.040*** | -0.496 | 0.247 | 1.195*** | 1.371*** | 1.550*** | 1.140*** |
|  | (0.396) | (0.173) | (0.217) | (0.078) | (0.474) | (0.300) | (0.072) | (0.257) | (0.246) | (0.072) |
| Observations | 614 | 3,345 | 893 | 6,541 | 582 | 939 | 2,997 | 768 | 688 | 1,917 |

**Notes:** * = p < 0.05, ** = p < 0.01, *** = p < 0.001

**Table A3. Poisson Regressions of HDDS 7 and Simpson’s Diversity at 25-km Scale**

|  | **Country Coef. (RSE)** | | | | | | | | | |
| --- | --- | --- | --- | --- | --- | --- | --- | --- | --- | --- |
| Variable | Benin | Burkina Faso | Cameroon | Ethiopia | Ghana | Guinea | Malawi | Nigeria | Uganda | Zimbabwe |
| Simpson's Diversity | 1.326*** | 0.189 | -1.101*** | -0.339*** | -0.591 | -0.356 | 0.093 | -0.788 | -0.115 | 0.456** |
|  | (0.311) | (0.240) | (0.208) | (0.071) | (0.715) | (0.561) | (0.129) | (0.602) | (0.199) | (0.154) |
| Sex of child = female | -0.023 | -0.051** | 0.080*** | 0.012 | -0.032 | 0.156*** | 0.006 | 0.054 | 0.045 | 0.034* |
|  | (0.024) | (0.020) | (0.022) | (0.014) | (0.034) | (0.033) | (0.014) | (0.033) | (0.028) | (0.015) |
| Head of household = female | -0.021 | 0.079* | -0.011 | -0.035 | 0.098* | -0.054 | -0.049** | 0.033 | -0.084* | 0.028 |
|  | (0.046) | (0.038) | (0.022) | (0.021) | (0.040) | (0.069) | (0.018) | (0.050) | (0.035) | (0.016) |
| Age of mother | 0.005** | 0.002 | -0.002 | -0.002 | 0.008*** | 0.006* | -0.002* | 0.002 | 0.000 | 0.002 |
|  | (0.002) | (0.001) | (0.002) | (0.001) | (0.002) | (0.003) | (0.001) | (0.002) | (0.002) | (0.001) |
| Age of child (ref = < 24 - 35 months) |  |  |  |  |  |  |  |  |  |  |
| 36 months - 47 months |  | -0.014 |  | 0.020 | -0.041 |  |  |  |  | -0.010 |
|  |  | (0.022) |  | (0.016) | (0.081) |  |  |  |  | (0.018) |
| 48 months - 59 months |  | 0.004 |  | -0.005 |  |  |  |  |  | 0.006 |
|  |  | (0.029) |  | (0.017) |  |  |  |  |  | (0.018) |
| Education of mother = primary school + | 0.030 | 0.175*** | -0.044 | 0.178*** | 0.134** | 0.099* | 0.170*** | 0.063 | 0.030 | 0.191*** |
|  | (0.031) | (0.031) | (0.040) | (0.019) | (0.043) | (0.039) | (0.017) | (0.037) | (0.039) | (0.028) |
| Rural = yes | 0.008 | -0.278*** | -0.052 | -0.162*** | 0.038 | -0.122 | -0.283*** | -0.112** | -0.225*** | -0.151** |
|  | (0.044) | (0.040) | (0.040) | (0.027) | (0.046) | (0.073) | (0.032) | (0.044) | (0.040) | (0.046) |
| Remoteness (ref = Q1) |  |  |  |  |  |  |  |  |  |  |
| Q2 | -0.023 | -0.027 | -0.008 | 0.031 | -0.108* | -0.481*** | 0.003 | 0.054 | 0.175*** | -0.081 |
|  | (0.052) | (0.043) | (0.044) | (0.037) | (0.051) | (0.073) | (0.034) | (0.054) | (0.042) | (0.046) |
| Q3 | 0.022 | 0.078 | 0.032 | -0.051 | -0.227** | -0.434*** | 0.046 | 0.041 | 0.149** | -0.096 |
|  | (0.060) | (0.046) | (0.050) | (0.039) | (0.072) | (0.070) | (0.037) | (0.064) | (0.055) | (0.049) |
| Q4 | 0.000 | -0.002 | -0.123* | -0.033 | -0.200** | -0.395*** | 0.068 | 0.210*** | 0.208*** | -0.132** |
|  | (0.058) | (0.051) | (0.056) | (0.039) | (0.069) | (0.081) | (0.038) | (0.061) | (0.055) | (0.050) |
| Q5 | 0.117 | 0.045 | -0.100 | -0.069 | -0.369*** | -0.466*** | 0.048 | 0.198* | 0.062 | -0.157** |
|  | (0.069) | (0.057) | (0.059) | (0.037) | (0.101) | (0.077) | (0.044) | (0.079) | (0.069) | (0.049) |
| Cropland, 10-km radius | -0.112 | 0.074 | -0.292* | 0.040 | 0.021 | 2.327*** | -0.092 | -0.214** | 0.015 | 0.253 |
|  | (0.107) | (0.139) | (0.115) | (0.066) | (0.108) | (0.460) | (0.074) | (0.075) | (0.062) | (0.146) |
| Pastureland, 10-km radius | -2.184*** | -0.165 | -0.454** | -0.163 | -0.087 | -0.478** | -0.014 | 0.161 | -0.240* | -0.091 |
|  | (0.598) | (0.121) | (0.167) | (0.089) | (0.154) | (0.167) | (0.056) | (0.129) | (0.096) | (0.047) |
| Annual Precipitation Change | -0.010* | -0.004 | 0.004** | 0.004*** | 0.016*** | 0.003* | 0.001 | 0.000 | 0.001 | -0.003** |
|  | (0.005) | (0.003) | (0.002) | (0.000) | (0.005) | (0.001) | (0.001) | (0.001) | (0.002) | (0.001) |
| Sample Region Fixed Effects | Yes | Yes | Yes | Yes | Yes | Yes | Yes | Yes | Yes | Yes |
| Constant | 1.648*** | 1.463*** | 1.897*** | 1.077*** | -0.142 | 0.253 | 1.190*** | 1.529*** | 1.476*** | 1.111*** |
|  | (0.385) | (0.178) | (0.249) | (0.083) | (0.627) | (0.365) | (0.087) | (0.308) | (0.241) | (0.084) |
| Observations | 614 | 3,345 | 893 | 6,541 | 582 | 939 | 2,997 | 768 | 688 | 1,917 |

**Notes:** * = p < 0.05, ** = p < 0.01, *** = p < 0.001

**Table A4. Poisson Regressions of HDDS and Simpson’s Diversity at 50-km Scale**

|  | **Country Coef. (RSE)** | | | | | | | | | |
| --- | --- | --- | --- | --- | --- | --- | --- | --- | --- | --- |
| Variable | Benin | Burkina Faso | Cameroon | Ethiopia | Ghana | Guinea | Malawi | Nigeria | Uganda | Zimbabwe |
| Simpson's Diversity | 0.407 | 0.423* | -1.069*** | -0.666*** | -0.529 | 2.553*** | 0.233 | -0.114 | 0.059 | 0.219 |
|  | (0.305) | (0.194) | (0.248) | (0.122) | (0.819) | (0.476) | (0.124) | (0.387) | (0.154) | (0.197) |
| Sex of child = female | -0.021 | -0.052** | 0.075*** | 0.010 | -0.033 | 0.121*** | 0.006 | 0.053 | 0.046 | 0.033* |
|  | (0.024) | (0.020) | (0.023) | (0.014) | (0.034) | (0.033) | (0.014) | (0.033) | (0.028) | (0.015) |
| Head of household = female | -0.020 | 0.077* | -0.011 | -0.033 | 0.099* | -0.052 | -0.050** | 0.031 | -0.090** | 0.027 |
|  | (0.047) | (0.038) | (0.022) | (0.020) | (0.040) | (0.068) | (0.018) | (0.050) | (0.034) | (0.016) |
| Age of mother | 0.004* | 0.002 | -0.001 | -0.002 | 0.008*** | 0.006 | -0.002* | 0.002 | 0.000 | 0.002 |
|  | (0.002) | (0.001) | (0.002) | (0.001) | (0.002) | (0.002) | (0.001) | (0.002) | (0.002) | (0.001) |
| Age of child (ref = < 24 - 35 months) |  |  |  |  |  |  |  |  |  |  |
| 36 months - 47 months |  | -0.015 |  | 0.020 | -0.037 |  |  |  |  | -0.007 |
|  |  | (0.022) |  | (0.016) | (0.084) |  |  |  |  | (0.018) |
| 48 months - 59 months |  | 0.003 |  | -0.005 |  |  |  |  |  | 0.006 |
|  |  | (0.029) |  | (0.017) |  |  |  |  |  | (0.018) |
| Education of mother = primary school + | 0.029 | 0.172*** | -0.041 | 0.175*** | 0.137** | 0.111** | 0.170*** | 0.064 | 0.032 | 0.195*** |
|  | (0.031) | (0.031) | (0.041) | (0.019) | (0.043) | (0.040) | (0.017) | (0.036) | (0.039) | (0.028) |
| Rural = yes | -0.013 | -0.282*** | -0.034 | -0.162*** | 0.033 | -0.014 | -0.282*** | -0.111* | -0.222*** | -0.165*** |
|  | (0.044) | (0.040) | (0.041) | (0.027) | (0.046) | (0.082) | (0.032) | (0.044) | (0.040) | (0.047) |
| Remoteness (ref = Q1) |  |  |  |  |  |  |  |  |  |  |
| Q2 | -0.006 | -0.030 | -0.028 | 0.027 | -0.102* | -0.503*** | -0.001 | 0.055 | 0.172*** | -0.073 |
|  | (0.053) | (0.042) | (0.045) | (0.036) | (0.052) | (0.078) | (0.034) | (0.054) | (0.042) | (0.047) |
| Q3 | 0.053 | 0.071 | 0.018 | -0.051 | -0.223** | -0.489*** | 0.037 | 0.039 | 0.133** | -0.088 |
|  | (0.060) | (0.046) | (0.050) | (0.038) | (0.073) | (0.074) | (0.037) | (0.063) | (0.051) | (0.050) |
| Q4 | -0.005 | -0.012 | -0.154** | -0.035 | -0.193** | -0.517*** | 0.056 | 0.221*** | 0.198*** | -0.115* |
|  | (0.060) | (0.051) | (0.058) | (0.038) | (0.069) | (0.082) | (0.039) | (0.061) | (0.053) | (0.051) |
| Q5 | 0.140 | 0.033 | -0.127* | -0.070 | -0.356*** | -0.482*** | 0.039 | 0.207** | 0.039 | -0.145** |
|  | (0.073) | (0.057) | (0.060) | (0.036) | (0.101) | (0.080) | (0.044) | (0.079) | (0.065) | (0.050) |
| Cropland, 10-km radius | -0.082 | 0.070 | -0.395*** | 0.071 | -0.012 | 0.478 | -0.089 | -0.227** | -0.002 | 0.389** |
|  | (0.110) | (0.141) | (0.118) | (0.066) | (0.107) | (0.512) | (0.070) | (0.075) | (0.057) | (0.138) |
| Pastureland, 10-km radius | -1.741** | -0.198 | -0.605*** | -0.155 | -0.071 | -0.279 | -0.026 | 0.141 | -0.271** | -0.078 |
|  | (0.562) | (0.118) | (0.177) | (0.088) | (0.154) | (0.168) | (0.056) | (0.128) | (0.100) | (0.049) |
| Annual Precipitation Change | -0.005 | -0.006* | 0.004** | 0.004*** | 0.015** | 0.003* | 0.001 | -0.001 | 0.000 | -0.002** |
|  | (0.004) | (0.003) | (0.002) | (0.000) | (0.005) | (0.001) | (0.001) | (0.001) | (0.002) | (0.001) |
| Sample Region Fixed Effects | Yes | Yes | Yes | Yes | Yes | Yes | Yes | Yes | Yes | Yes |
| Constant | 1.629*** | 1.487*** | 1.899*** | 1.298*** | -0.140 | -0.709* | 1.104*** | 1.238*** | 1.421*** | 1.169*** |
|  | (0.435) | (0.175) | (0.262) | (0.107) | (0.713) | (0.321) | (0.090) | (0.260) | (0.252) | (0.110) |
| Observations | 614 | 3,345 | 893 | 6,541 | 582 | 939 | 2,997 | 768 | 688 | 1,917 |

**Notes:** * = p < 0.05, ** = p < 0.01, *** = p < 0.001

**Table A5. Simpson’s Diversity Index Across Samples**

|  | **Scale of Buffer** | | | | | | | | | | | | |
| --- | --- | --- | --- | --- | --- | --- | --- | --- | --- | --- | --- | --- | --- |
|  | **10-km** | | | | **25-km** | | | | **50-km** | | | | |
| **Sample** | **Mean** | **SD** | **Min** | **Max** | **Mean** | **SD** | **Min** | **Max** | **Mean** | **SD** | **Min** | **Max** |  |
| Benin 2001 | 0.430 | 0.092 | 0.141 | 0.640 | 0.534 | 0.063 | 0.394 | 0.680 | 0.606 | 0.065 | 0.461 | 0.699 |  |
| Burkina Faso 2003 | 0.257 | 0.051 | 0.054 | 0.457 | 0.321 | 0.062 | 0.202 | 0.601 | 0.372 | 0.075 | 0.233 | 0.636 |  |
| Cameroon 2004 | 0.416 | 0.138 | 0.000 | 0.681 | 0.496 | 0.118 | 0.000 | 0.708 | 0.559 | 0.091 | 0.000 | 0.727 |  |
| Ethiopia 2000 | 0.423 | 0.155 | 0.000 | 0.656 | 0.532 | 0.136 | 0.000 | 0.713 | 0.613 | 0.072 | 0.053 | 0.716 |  |
| Ghana 2003 | 0.453 | 0.076 | 0.156 | 0.570 | 0.512 | 0.046 | 0.366 | 0.615 | 0.548 | 0.037 | 0.448 | 0.637 |  |
| Guinea 2005 | 0.330 | 0.086 | 0.000 | 0.472 | 0.378 | 0.055 | 0.201 | 0.571 | 0.421 | 0.058 | 0.352 | 0.584 |  |
| Malawi 2000 | 0.426 | 0.098 | 0.000 | 0.630 | 0.511 | 0.073 | 0.233 | 0.697 | 0.594 | 0.069 | 0.394 | 0.744 |  |
| Malawi 2004 | 0.428 | 0.100 | 0.000 | 0.657 | 0.510 | 0.075 | 0.233 | 0.679 | 0.591 | 0.066 | 0.364 | 0.744 |  |
| Nigeria 2003 | 0.455 | 0.067 | 0.000 | 0.618 | 0.482 | 0.031 | 0.380 | 0.669 | 0.505 | 0.041 | 0.422 | 0.689 |  |
| Uganda 2001 | 0.459 | 0.086 | 0.000 | 0.785 | 0.513 | 0.100 | 0.000 | 0.807 | 0.559 | 0.120 | 0.000 | 0.810 |  |
| Zimbabwe 1999 | 0.303 | 0.073 | 0.000 | 0.707 | 0.376 | 0.067 | 0.185 | 0.774 | 0.433 | 0.067 | 0.323 | 0.793 |  |

**Tables B**

**Table B1. Poisson Regressions of HDDS and Simpson’s Diversity at 10-km Scale**

|  | **Country Coef. (RSE)** | | | | | | | | | |
| --- | --- | --- | --- | --- | --- | --- | --- | --- | --- | --- |
| Variable | Benin | Burkina Faso | Cameroon | Ethiopia | Ghana | Guinea | Malawi | Nigeria | Uganda | Zimbabwe |
| Simpson's Diversity | 0.047 | 0.9578*** | -0.0912 | -0.3812*** | 0.4226 | -1.1238*** | 0.095 | -0.2273 | 0.07 | 0.4835*** |
|  | (0.211) | (0.239) | (0.076) | (0.059) | (0.251) | (0.229) | (0.063) | (0.319) | (0.122) | (0.127) |
| Sex of child = female | -0.003 | 0.0044 | 0.0939*** | -0.009 | -0.0201 | 0.0654** | 0.0014 | 0.0626** | -0.0331* | 0.0243 |
|  | (0.019) | (0.016) | (0.013) | (0.012) | (0.023) | (0.025) | (0.009) | (0.021) | (0.016) | (0.013) |
| Head of household = female | 0.006 | 0.0246 | -0.0232 | -0.0564** | -0.0295 | -0.1562*** | -0.0465*** | 0.1154** | -0.0911*** | -0.0108 |
|  | (0.032) | (0.035) | (0.017) | (0.018) | (0.028) | (0.046) | (0.011) | (0.038) | (0.024) | (0.013) |
| Age of mother | 0.0011 | 0.002 | 0.0007 | -0.0011 | 0.002 | 0.0055*** | -0.0002 | 0 | 0.0012 | 0.0012 |
|  | (0.001) | (0.001) | (0.001) | (0.001) | (0.002) | (0.002) | (0.001) | (0.001) | (0.001) | (0.001) |
| Age of child (ref = < 6 - 11 months) |  |  |  |  |  |  |  |  |  |  |
| 12 months - 23 months | 0.439*** | 0.2067*** | 0.2002*** | 0.2798*** | 0.2644*** | 0.1631*** | 0.2399*** | 0.2855*** | 0.1974*** | 0.34*** |
|  | (0.035) | (0.032) | (0.021) | (0.029) | (0.038) | (0.037) | (0.014) | (0.032) | (0.023) | (0.030) |
| 24 months - 35 months | 0.597*** | 0.3059*** | 0.2616 | 0.3193 | 0.3366*** | 0.2987*** | 0.3066*** | 0.4395*** | 0.2006*** | 0.3957*** |
|  | (0.035) | (0.031) | (0.022)*** | (0.029)*** | (0.039) | (0.038) | (0.015) | (0.032) | (0.026) | (0.029) |
| 36 months - 47 months |  | 0.2954*** |  | 0.3377*** | 0.3801*** |  |  |  |  | 0.3864*** |
|  |  | (0.033) |  | (0.029) | (0.087) |  |  |  |  | (0.031) |
| 48 months - 59 months |  | 0.3128*** |  | 0.3132*** |  |  |  |  |  | 0.3975*** |
|  |  | (0.038) |  | (0.030) |  |  |  |  |  | (0.030) |
| Education of mother = primary school + | 0.0519* | 0.1967 | -0.0289 | 0.1828 | 0.1571*** | 0.0722* | 0.1177 | 0.0982*** | 0.1247*** | 0.1729*** |
|  | (0.024) | (0.026) | (0.024) | (0.016) | (0.027) | (0.036) | (0.011) | (0.025) | (0.021) | (0.024) |
| Rural = yes | -0.1*** | -0.1962 | -0.0881*** | -0.1746*** | -0.0389 | -0.0796 | -0.2131 | -0.1842*** | -0.11*** | -0.064 |
|  | (0.028) | (0.029) | (0.023) | (0.025) | (0.034) | (0.049) | (0.024) | (0.029) | (0.025) | (0.043) |
| Remoteness (ref = Q1) |  |  |  |  |  |  |  |  |  |  |
| Q2 | -0.0816* | 0.0063 | 0.0092 | 0.0122 | 0.0049 | -0.3254*** | 0.0239 | -0.03 | 0.0345 | -0.1215** |
|  | (0.038) | (0.031) | (0.027) | (0.033) | (0.038) | (0.060) | (0.026) | (0.034) | (0.026) | (0.043) |
| Q3 | -0.0366 | -0.0003 | 0.0288 | -0.0542 | -0.09 | -0.1891*** | 0.0591* | -0.0652 | 0.0571 | -0.139** |
|  | (0.045) | (0.034) | (0.030) | (0.036) | (0.048) | (0.056) | (0.024) | (0.040) | (0.032) | (0.045) |
| Q4 | -0.0653 | -0.0324 | -0.0661* | -0.0363 | -0.1038 | -0.1566** | 0.0198 | 0.1085* | 0.0315 | -0.1391** |
|  | (0.049) | (0.038) | (0.033) | (0.036) | (0.052) | (0.059) | (0.024) | (0.042) | (0.034) | (0.046) |
| Q5 | -0.0405 | 0.0359 | -0.0913** | -0.0866* | -0.136* | -0.2876*** | 0.0015 | -0.0673 | 0.0345 | -0.2074*** |
|  | (0.052) | (0.041) | (0.035) | (0.034) | (0.070) | (0.071) | (0.027) | (0.057) | (0.040) | (0.046) |
| Cropland, 10-km radius | -0.0002 | 0.062 | -0.3465*** | 0.1847** | -0.0462 | 0.8053** | -0.0365 | -0.0692 | -0.0884* | 0.2287 |
|  | (0.090) | (0.131) | (0.069) | (0.065) | (0.092) | (0.300) | (0.054) | (0.053) | (0.039) | (0.132) |
| Pastureland, 10-km radius | -1.4961*** | -0.1984* | 0.453*** | -0.2306*** | -0.109 | -0.8454*** | -0.0639 | 0.115 | -0.1006 | -0.0943* |
|  | (0.290) | (0.092) | (0.089) | (0.076) | (0.105) | (0.105) | (0.034) | (0.089) | (0.056) | (0.039) |
| Annual Precipitation Change | -0.0179*** | -0.005* | 0.0043*** | 0.0031*** | 0.0076* | -0.0004 | 0.0011* | -0.0001 | 0.0026* | -0.0015* |
|  | (0.004) | (0.002) | (0.001) | (0.000) | (0.003) | (0.001) | (0.001) | (0.001) | (0.001) | (0.001) |
| Sample Region Fixed Effects | Yes | Yes | Yes | Yes | Yes | Yes | Yes | Yes | Yes | Yes |
| Constant | 2.4285*** | 0.9987*** | 0.9284*** | 0.7605*** | -0.1217*** | 1.0558*** | 0.8138*** | 0.9496*** | 0.9482*** | 0.7681*** |
|  | (0.349) | (0.138) | (0.124) | (0.067) | (0.313) | (0.182) | (0.050) | (0.150) | (0.138) | (0.068) |
| Observations | 1,817 | 5,698 | 3,212 | 9,244 | 1,576 | 2,275 | 9,871 | 2,386 | 2,514 | 3,092 |

**Notes:** * = p < 0.05, ** = p < 0.01, *** = p < 0.001

**Table B2. Poisson Regressions of HDDS and Simpson’s Diversity at 25-km Scale**

|  | **Country Coef. (RSE)** | | | | | | | | | |
| --- | --- | --- | --- | --- | --- | --- | --- | --- | --- | --- |
| Variable | Benin | Burkina Faso | Cameroon | Ethiopia | Ghana | Guinea | Malawi | Nigeria | Uganda | Zimbabwe |
| Simpson's Diversity | 0.971*** | 0.589** | -0.556*** | -0.364*** | -0.228 | -1.486*** | 0.317*** | 0.107 | -0.206 | 0.435*** |
|  | (0.209) | (0.185) | (0.075) | (0.061) | (0.440) | (0.330) | (0.086) | (0.345) | (0.112) | (0.130) |
| Sex of child = female | -0.008 | 0.006 | 0.093*** | -0.010 | -0.020 | 0.078** | 0.002 | 0.062** | -0.034* | 0.027* |
|  | (0.019) | (0.016) | (0.013) | (0.012) | (0.023) | (0.025) | (0.009) | (0.021) | (0.016) | (0.013) |
| Head of household = female | 0.010 | 0.023 | -0.023 | -0.056** | -0.028 | -0.147** | -0.047*** | 0.116** | -0.088*** | -0.008 |
|  | (0.032) | (0.034) | (0.017) | (0.018) | (0.028) | (0.045) | (0.011) | (0.038) | (0.024) | (0.013) |
| Age of mother | 0.001 | 0.002 | 0.000 | -0.001 | 0.002 | 0.006*** | 0.000 | 0.000 | 0.001 | 0.001 |
|  | (0.001) | (0.001) | (0.001) | (0.001) | (0.002) | (0.002) | (0.001) | (0.001) | (0.001) | (0.001) |
| Age of child (ref = < 6 - 11 months) |  |  |  |  |  |  |  |  |  |  |
| 12 months - 23 months | 0.442*** | 0.209*** | 0.200*** | 0.278*** | 0.264*** | 0.167*** | 0.241*** | 0.284*** | 0.198*** | 0.340*** |
|  | (0.035) | (0.032) | (0.020) | (0.029) | (0.038) | (0.037) | (0.014) | (0.032) | (0.023) | (0.030) |
| 24 months - 35 months | 0.601*** | 0.311*** | 0.264*** | 0.320*** | 0.335*** | 0.300*** | 0.307*** | 0.440*** | 0.200*** | 0.398*** |
|  | (0.034) | (0.031) | (0.021) | (0.029) | (0.039) | (0.038) | (0.015) | (0.032) | (0.026) | (0.030) |
| 36 months - 47 months |  | 0.298*** |  | 0.339*** | 0.370 |  |  |  |  | 0.388*** |
|  |  | (0.032) |  | (0.029) | (0.082) |  |  |  |  | (0.031) |
| 48 months - 59 months |  | 0.314*** |  | 0.313*** |  |  |  |  |  | 0.398*** |
|  |  | (0.038) |  | (0.030) |  |  |  |  |  | (0.030) |
| Education of mother = primary school + | 0.053* | 0.202 | -0.043 | 0.185*** | 0.159*** | 0.059 | 0.117*** | 0.098*** | 0.120*** | 0.176*** |
|  | (0.024) | (0.026) | (0.024) | (0.016) | (0.027) | (0.037) | (0.011) | (0.025) | (0.021) | (0.024) |
| Rural = yes | -0.082** | -0.205 | -0.100*** | -0.172*** | -0.036 | -0.125* | -0.212*** | -0.185*** | -0.117*** | -0.066 |
|  | (0.028) | (0.029) | (0.023) | (0.024) | (0.034) | (0.049) | (0.024) | (0.030) | (0.025) | (0.043) |
| Remoteness (ref = Q1) |  |  |  |  |  |  |  |  |  |  |
| Q2 | -0.093* | -0.005 | 0.012 | 0.005 | 0.006 | -0.315*** | 0.020 | -0.029 | 0.040 | -0.130** |
|  | (0.037) | (0.031) | (0.027) | (0.033) | (0.038) | (0.061) | (0.026) | (0.034) | (0.026) | (0.043) |
| Q3 | -0.050 | -0.008 | 0.039 | -0.063 | -0.086 | -0.151** | 0.047* | -0.065 | 0.081* | -0.132** |
|  | (0.045) | (0.034) | (0.030) | (0.036) | (0.048) | (0.056) | (0.024) | (0.040) | (0.033) | (0.045) |
| Q4 | -0.050 | -0.040 | -0.053 | -0.050 | -0.108* | -0.129* | 0.005 | 0.112** | 0.047 | -0.144** |
|  | (0.046) | (0.038) | (0.033) | (0.035) | (0.052) | (0.059) | (0.024) | (0.042) | (0.035) | (0.046) |
| Q5 | -0.025 | 0.024 | -0.081* | -0.091 | -0.140* | -0.275*** | -0.020 | -0.060 | 0.068 | -0.217*** |
|  | (0.051) | (0.041) | (0.035) | (0.034) | (0.071) | (0.071) | (0.027) | (0.057) | (0.043) | (0.046) |
| Cropland, 10-km radius | -0.058 | 0.217 | -0.326*** | 0.092** | 0.045 | 1.037** | -0.042 | -0.089 | -0.065* | 0.401** |
|  | (0.079) | (0.119) | (0.064) | (0.057) | (0.078) | (0.351) | (0.047) | (0.048) | (0.033) | (0.122) |
| Pastureland, 10-km radius | -1.794*** | -0.179 | 0.436*** | -0.229* | -0.131 | -0.914*** | -0.087* | 0.091 | -0.077 | -0.096* |
|  | (0.285) | (0.095) | (0.089) | (0.076) | (0.107) | (0.103) | (0.034) | (0.089) | (0.056) | (0.039) |
| Annual Precipitation Change | -0.020*** | -0.004 | 0.004*** | 0.003*** | 0.007* | -0.001 | 0.001** | 0.000 | 0.003* | -0.001 |
|  | (0.004) | (0.002) | (0.001) | (0.000) | (0.003) | (0.001) | (0.001) | (0.001) | (0.001) | (0.001) |
| Sample Region Fixed Effects | Yes | Yes | Yes | Yes | Yes | Yes | Yes | Yes | Yes | Yes |
| Constant | 2.158*** | 0.970*** | 1.249*** | 0.799*** | 0.248 | 1.307*** | 0.699*** | 0.822*** | 1.029*** | 0.719*** |
|  | (0.357) | (0.141) | (0.131) | (0.073) | (0.390) | (0.192) | (0.058) | (0.184) | (0.140) | (0.077) |
| Observations | 1,817 | 5,698 | 3,212 | 9,244 | 1,576 | 2,275 | 9,871 | 2,386 | 2,514 | 3,092 |

**Notes:** * = p < 0.05, ** = p < 0.01, *** = p < 0.001

**Table B3. Poisson Regressions of HDDS and Simpson’s Diversity at 50-km Scale**

|  | **Country Coef. (RSE)** | | | | | | | | | |
| --- | --- | --- | --- | --- | --- | --- | --- | --- | --- | --- |
| Variable | Benin | Burkina Faso | Cameroon | Ethiopia | Ghana | Guinea | Malawi | Nigeria | Uganda | Zimbabwe |
| Simpson's Diversity | 0.308 | 0.336* | -0.084 | -0.635*** | -0.116 | 0.584 | 0.263*** | 0.480* | -0.078 | 0.081 |
|  | (0.232) | (0.160) | (0.120) | (0.111) | (0.500) | (0.351) | (0.077) | (0.224) | (0.090) | (0.151) |
| Sex of child = female | -0.004 | 0.004 | 0.094*** | -0.011 | -0.020 | 0.063* | 0.001 | 0.064** | -0.034* | 0.026* |
|  | (0.019) | (0.016) | (0.013) | (0.012) | (0.023) | (0.025) | (0.009) | (0.021) | (0.016) | (0.013) |
| Head of household = female | 0.006 | 0.023 | -0.023 | -0.054** | -0.028 | -0.140** | -0.048*** | 0.117** | -0.089*** | -0.010 |
|  | (0.032) | (0.034) | (0.017) | (0.018) | (0.028) | (0.045) | (0.011) | (0.039) | (0.024) | (0.013) |
| Age of mother | 0.001 | 0.002 | 0.001 | -0.001 | 0.002 | 0.005*** | 0.000 | 0.000 | 0.001 | 0.001 |
|  | (0.001) | (0.001) | (0.001) | (0.001) | (0.002) | (0.002) | (0.001) | (0.001) | (0.001) | (0.001) |
| Age of child (ref = < 6 - 11 months) |  |  |  |  |  |  |  |  |  |  |
| 12 months - 23 months | 0.441*** | 0.210*** | 0.200*** | 0.278*** | 0.264*** | 0.146*** | 0.240*** | 0.284*** | 0.199*** | 0.339*** |
|  | (0.035) | (0.032) | (0.021) | (0.029) | (0.038) | (0.037) | (0.014) | (0.032) | (0.024) | (0.030) |
| 24 months - 35 months | 0.598*** | 0.315*** | 0.261*** | 0.316*** | 0.335*** | 0.286*** | 0.306*** | 0.443*** | 0.202*** | 0.396*** |
|  | (0.034) | (0.031) | (0.022) | (0.029) | (0.038) | (0.038) | (0.015) | (0.032) | (0.026) | (0.029) |
| 36 months - 47 months |  | 0.302*** |  | 0.336*** | 0.371 |  |  |  |  | 0.389*** |
|  |  | (0.032) |  | (0.029) | (0.083) |  |  |  |  | (0.031) |
| 48 months - 59 months |  | 0.317*** |  | 0.309*** |  |  |  |  |  | 0.398*** |
|  |  | (0.038) |  | (0.030) |  |  |  |  |  | (0.030) |
| Education of mother = primary school + | 0.052* | 0.201 | -0.027 | 0.183 | 0.159*** | 0.065 | 0.118*** | 0.096*** | 0.123*** | 0.176*** |
|  | (0.024) | (0.026) | (0.023) | (0.016) | (0.028) | (0.036) | (0.011) | (0.026) | (0.021) | (0.025) |
| Rural = yes | -0.097*** | -0.207 | -0.084*** | -0.173 | -0.036 | -0.116 | -0.213*** | -0.184*** | -0.114*** | -0.079 |
|  | (0.028) | (0.029) | (0.023) | (0.024) | (0.034) | (0.051) | (0.024) | (0.030) | (0.025) | (0.044) |
| Remoteness (ref = Q1) |  |  |  |  |  |  |  |  |  |  |
| Q2 | -0.080* | -0.009 | 0.007 | 0.003 | 0.007 | -0.307*** | 0.018 | -0.030 | 0.035 | -0.121** |
|  | (0.037) | (0.031) | (0.027) | (0.033) | (0.038) | (0.062) | (0.026) | (0.034) | (0.026) | (0.044) |
| Q3 | -0.033 | -0.012 | 0.029 | -0.060 | -0.086 | -0.182** | 0.049* | -0.073 | 0.065* | -0.127** |
|  | (0.045) | (0.034) | (0.030) | (0.035) | (0.048) | (0.058) | (0.024) | (0.040) | (0.032) | (0.046) |
| Q4 | -0.059 | -0.051 | -0.064 | -0.048 | -0.106* | -0.158* | 0.007 | 0.106* | 0.036 | -0.130** |
|  | (0.047) | (0.038) | (0.033) | (0.035) | (0.052) | (0.062) | (0.025) | (0.043) | (0.034) | (0.046) |
| Q5 | -0.020 | 0.023 | -0.095** | -0.091** | -0.140* | -0.268*** | -0.008 | -0.066 | 0.044 | -0.207*** |
|  | (0.055) | (0.042) | (0.035) | (0.033) | (0.071) | (0.071) | (0.027) | (0.057) | (0.041) | (0.047) |
| Cropland, 10-km radius | -0.004 | 0.289* | -0.377*** | 0.096 | 0.033 | -0.121 | -0.010 | -0.100* | -0.075* | 0.534*** |
|  | (0.078) | (0.120) | (0.064) | (0.056) | (0.074) | (0.373) | (0.045) | (0.048) | (0.032) | (0.117) |
| Pastureland, 10-km radius | -1.568*** | -0.136 | 0.444*** | -0.229** | -0.121 | -0.903*** | -0.085* | 0.081 | -0.080 | -0.074 |
|  | (0.283) | (0.093) | (0.089) | (0.076) | (0.106) | (0.105) | (0.035) | (0.089) | (0.059) | (0.040) |
| Annual Precipitation Change | -0.017*** | -0.003 | 0.004*** | 0.003*** | 0.007* | -0.002* | 0.001* | 0.000 | 0.003* | -0.001 |
|  | (0.004) | (0.002) | (0.001) | (0.000) | (0.003) | (0.001) | (0.001) | (0.001) | (0.001) | (0.001) |
| Sample Region Fixed Effects | Yes | Yes | Yes | Yes | Yes | Yes | Yes | Yes | Yes | Yes |
| Constant | 2.226*** | 0.950*** | 0.955*** | 0.982*** | 0.182 | 0.849*** | 0.711*** | 0.627*** | 1.016*** | 0.830*** |
|  | (0.396) | (0.141) | (0.146) | (0.097) | (0.427) | (0.206) | (0.060) | (0.155) | (0.146) | (0.091) |
| Observations | 1,817 | 5,698 | 3,212 | 9,244 | 1,576 | 2,275 | 9,871 | 2,386 | 2,514 | 3,092 |

**Notes:** * = p < 0.05, ** = p < 0.01, *** = p < 0.001
